# Cost per DALY averted in low, middle- and high-income countries: evidence from the global burden of disease study to estimate the cost-effectiveness thresholds

**DOI:** 10.1186/s12962-021-00260-0

**Published:** 2021-02-04

**Authors:** Rajabali Daroudi, Ali Akbari Sari, Azin Nahvijou, Ahmad Faramarzi

**Affiliations:** 1 Department of Health Management and Economics, School of Public Health, Tehran University of Medical Sciences, Tehran, Iran; 2 Cancer Research Center, Cancer Institute of Iran, Tehran University of Medical Sciences, Tehran, Iran; 3 Department of Health Management and Economics, School of Public Health, Urmia University of Medical Sciences, Urmia, Iran

**Keywords:** DALY, HDI, GDP per capita, Health expenditure

## Abstract

**Background:**

Determining the cost-effectiveness thresholds for healthcare interventions has been a severe challenge for policymakers, especially in low- and middle-income countries. This study aimed to estimate the cost per disability-adjusted life-year (DALY) averted for countries with different levels of Human Development Index (HDI) and Gross Domestic Product (GDP).

**Methods:**

The data about DALYs, per capita health expenditure (HE), HDI, and GDP per capita were extracted for 176 countries during the years 2000 to 2016. Then we examined the trends on these variables. Panel regression analysis was performed to explore the correlation between DALY and HE per capita. The results of the regression models were used to calculate the cost per DALY averted for each country.

**Results:**

Age-standardized rate (ASR) DALY (DALY per 100,000 population) had a nonlinear inverse correlation with HE per capita and a linear inverse correlation with HDI. One percent increase in HE per capita was associated with an average of 0.28, 0.24, 0.18, and 0.27% decrease on the ASR DALY in low HDI, medium HDI, high HDI, and very high HDI countries, respectively. The estimated cost per DALY averted was $998, $6522, $23,782, and $69,499 in low HDI, medium HDI, high HDI, and very high HDI countries. On average, the cost per DALY averted was 0.34 times the GDP per capita in low HDI countries. While in medium HDI, high HDI, and very high HDI countries, it was 0.67, 1.22, and 1.46 times the GDP per capita, respectively.

**Conclusions:**

This study suggests that the cost-effectiveness thresholds might be less than a GDP per capita in low and medium HDI countries and between one and two GDP per capita in high and very high HDI countries.

## Background

Policymakers always need effective mechanisms for allocating limited healthcare resources among different healthcare interventions [1]. Cost-effectiveness analysis (CEA) is an essential and commonly used approach for such priority setting in healthcare. CEA results are often expressed as an incremental cost-effectiveness ratio (ICER), the ratio of incremental costs to incremental outcomes. In CEA studies, generic measures such as quality-adjusted life-years (QALYs) and disability-adjusted life-years (DALYs) are commonly used to measure effectiveness. The ICER provides cost per QALY gained or cost per DALY averted compared to the next best alternative. To determine whether a new intervention is cost-effective, we usually need to compare its ICER with a benchmark or cost-effectiveness threshold. If the ICER (cost per QALY gained or cost per DALY averted) falls below the defined threshold, the intervention is considered cost-effective. If the ICER is above the threshold, the intervention is not considered cost-effective [2, 3].

Determining the cost-effectiveness thresholds for healthcare interventions has been the topic of controversy and a significant challenge for policymakers, including prioritizing health intervention, deciding to include new interventions, and deciding how to allocate resources [4–6]. Generally, there are two approaches, including demand-side and supply-side approaches, for estimating the cost-effectiveness thresholds of healthcare interventions [3, 7]. In the demand-side approach, the social monetary value of a QALY is usually estimated through a willingness to pay survey in a representative sample of the general population. Thus the estimated monetary value for a QALY can be used as a threshold [3].

In the supply-side approach, the threshold is determined to reflect the opportunity cost of spending on health by linking the healthcare expenditure to health outcomes. In this approach, an intervention is considered cost-effective if its health generated is higher than the health that could have been produced on other interventions with the same money spent. So the threshold is the ICER of the least cost-effective currently funded intervention [3, 8].

According to previous studies, thresholds that are defined based on the demand-side approaches are generally higher than thresholds that are estimated from the supply-side approaches [7, 9]. It is argued that in situations where the health system budget can be increased, the demand-side thresholds can be used suitably. However, in situations where the health system budget is constrained and cannot be increased, using these thresholds may lead to decisions that reduce rather than improve health outcomes. Because in these situations, a new intervention can be funded only by replacing previous interventions, and the health outcome of the new intervention may be less than the omitted interventions. Moreover, since the supply-side thresholds are based on the opportunity cost to the healthcare system, by using these thresholds, the aggregate health is improved by the inclusion of new interventions [3, 10].

In recent years, several studies have been conducted to estimate cost-effectiveness thresholds worldwide [7, 8]. However, most of these studies have been conducted in high-income countries and have used demand-side approaches. There is little evidence about opportunity-cost-base thresholds, especially in low- and middle-income countries (LMICs). Using the global burden of disease data in this study, we aimed to estimate the cost per DALY averted for countries with different levels of Human Development Index (HDI) and Gross Domestic Product (GDP) per capita. The results could help health policymakers in different countries to determine appropriate cost-effectiveness thresholds and make better decisions.

## Methods

### Data

The data about DALY, per capita HE, HDI, and per capita GDP were extracted for 176 countries from 2000 to 2016. The data on DALYs were retrospectively extracted from the Global Burden of Disease (GBD) study, which is published by the Institute for Health Metrics and Evaluation (IHME) [11]. DALY numbers, crude DALY rates, and age-standardized DALY rates were extracted for each country. Data on HE per capita (PPP constant 2011 INT$) and GDP per capita (PPP $ 2011) were extracted from World Bank databases [12]. Data on HDI were taken from the United Nations Development Program [13].

### Statistical analysis

We used charts to illustrate the global trends on DALY and HE per capita from 2000 to 2016. Also, the charts were useful to reveal the relationships between variables. Using the box plot chart, we drew the relationships between the ASR DALY (per 100,000 population), HE per capita, and HDI of each country in 2016. According to HDI, countries were categorized into four groups, including low HDI, medium HDI, high, and very high HDI. Then, the DALY and HE per capita trend in each group was explored during the years 2000 to 2016.

Besides, panel regression analysis was performed to assess the relationship between DALY and HE per capita. We used the natural logarithm of the ASR DALY (per 100,000 population) and HE per capita (PPP constant 2011 INT$), then estimated the following regression model:

$${\text{logDALY}}_{{{\text{it}}}} = \alpha + \beta {\text{logHE}} + \varepsilon_{{{\text{it}}}}$$

In this model, logDALY_it_ represented natural logarithm of the ASR DALY (per 100,000 population) for country i and year t, logHE represented the natural logarithm of HE per capita (PPP constant 2011 INT$), β is the vector of regression coefficients and shows the percent change in ASR DALY (per 100,000 population) due to 1% change in the HE per capita (elasticity of ASR DALY to HE per capita), and ε_it_ is the error term. This model was estimated for each HDI category separately as well as for all countries together.

The Hausman test results indicated that the fixed-effects model would perform better and was subsequently used in all regression models. P values < 0.05 were considered significant.

### Cost per DALY averted

Using the results of the regression models, we calculated the cost per DALY averted for each country. Since the regression models were log–log models, we used the following formula to calculate the cost per DALY averted for each country.

$$cost\, per\, DALY\, averted=\frac{0.01*{\text{HE per capita }}\left({\text{PPP constant }} 2011 {\text{ INT}}\$\right)}{\beta *{\text{ASR DALY per capita}}}$$

As mentioned before, β is the elasticity of ASR DALY to HE per capita and varies by the HDI category. So for countries in each HDI category, the β for that category was used. Since HE per capita and ASR DALY vary in different years for a country, the cost per DALY averted would not be the same in different years. In this study, we calculated the cost per DALY averted based on data in 2016. Furthermore, we used a scatter plot to show the relationship between the costs per DALY averted and GDP per capita in 2016.

Regression analyses were performed using Stata 14 (Stata Corp, College Station, Tex) software. We used Microsoft Office Excel 2013 (Microsoft, Redmond, WA) to perform other analyses and drawing Charts.

## Results

### Global trends for DALY and health expenditure per capita

**Fig. 1:**
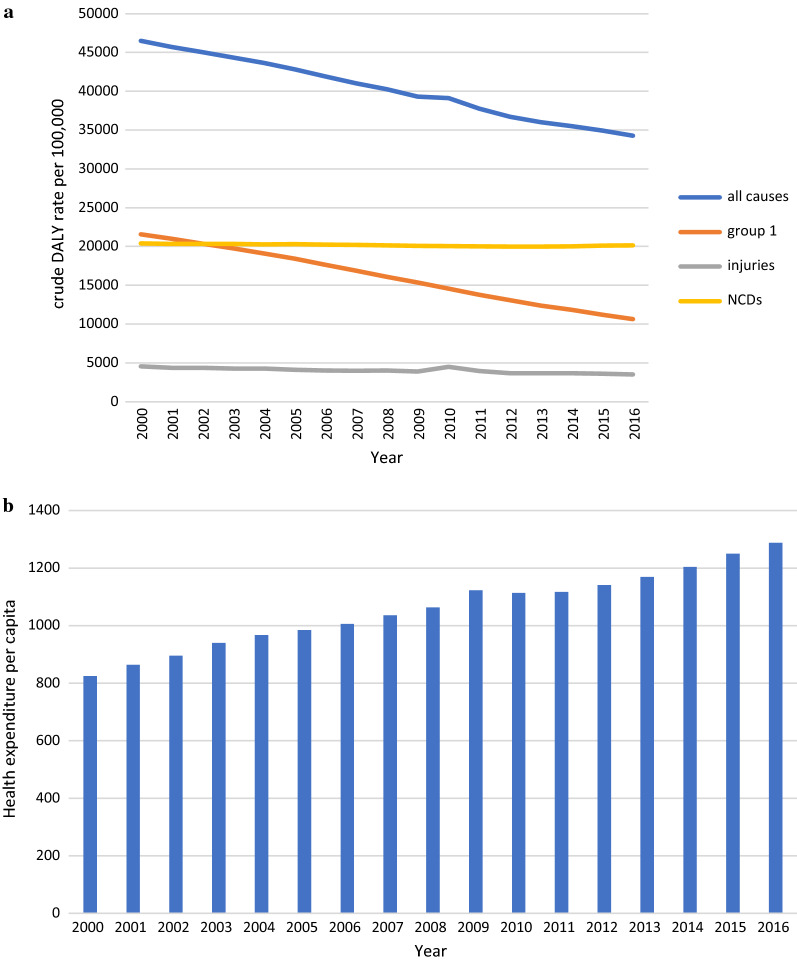
**a** Trend of crude DALY rate per 100,000 based on the diseased group from 2000 to 2016, group 1 included communicable, maternal, neonatal and nutritional diseases, NCDs (non-communicable disease). **b** The mean of health expenditure per capita (PPP $2011 international) from 2000 to 2016 in 176 countries

Figure 1 shows the DALY rates based on diseased groups and health expenditure per capita from 2000 to 2016 for 176 countries. DALY rates due to all causes demonstrated a sustained decline from 2000 to 2016 in 176 countries. Crude DALY rates for all causes were 46,495 per 100,000 population in 2000, which decreased to 34,280 in 2016. The trend due to group 1 causes decreased similarly, from 21,560 per 100,000 in 2000 to 10,642 in 2016. The DALY rate trend due to NCDs was almost constant from 2000 to 2016, whereas the trend related to injuries has not been entirely constant, with a maximum of 4496 in 2010.

The mean of health expenditure per capita (PPP 2011$) for 176 countries was $824 in 2000, and this value increased to 2016 at $1288.

### Correlation of DALYs with health expenditure and human development index

**Fig. 2:**
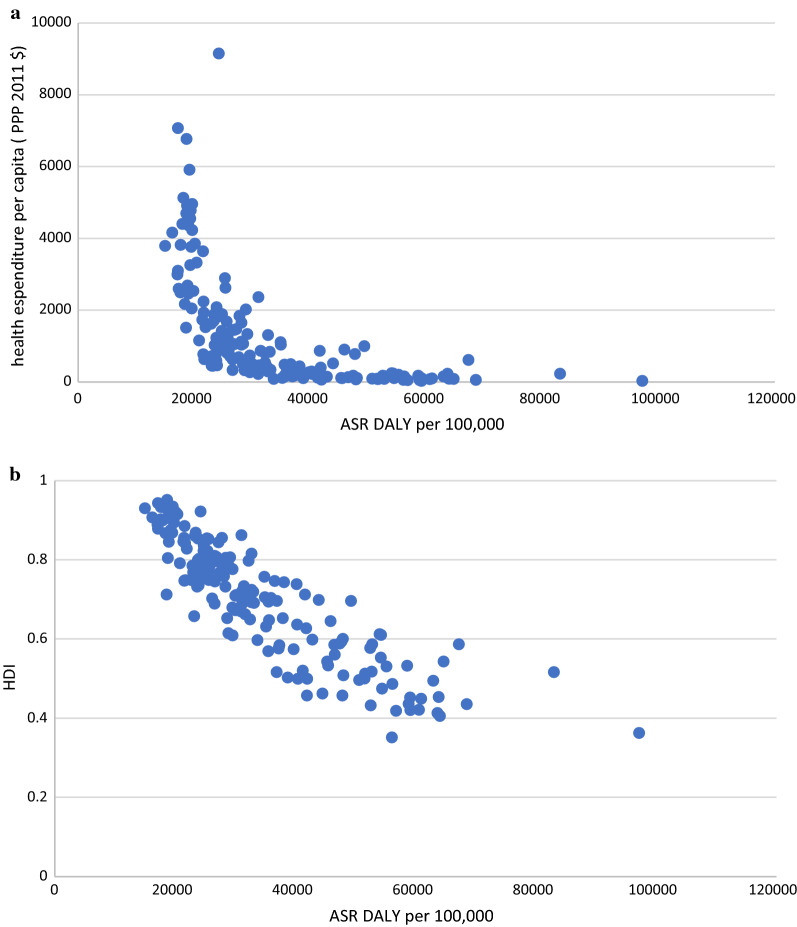
**a** Correlation between health expenditure per capita (PPP $2011 international) and ASR DALY due to all causes in 2016. **b** Correlation between Human Development Index (HDI) and ASR DALY due to all causes in 2016

Distinct patterns emerged for DALY rate and health expenditure per capita (Fig. 2). There was a nonlinear inverse relationship between health expenditure per capita and ASR DALY (per 100,000 population). As HE per capita (PPP constant 2011 INT$) increases, the ASR DALY (per 100,000 population) reduces with a decreasing rate. However, some countries were almost similar in terms of ASR DALY but were relatively different in HE per capita or vice versa. For example, Portugal and Australia were nearly the same in terms of ASR DALY (18,962 vs 18,967 years), but they were different in HE per capita ($2458 vs $4367). Like this, the United States and Lebanon had nearly the same ASR DALY (24,235 vs 24,383 years), while their HE per capita was quite different ($9151 vs $1063).

Figure 2 shows a reverse linear relationship between HDI and ASR DALY rate due to all causes. Overall, countries with higher HDI have lower ASR DALY rates. For example, Central Africa Republic, Chad, Sierra Leone, Lesotho, Niger, Guinea, Mali, Mozambique, Malawi, Burundi, and Liberia have low HDI and high ASR DALY rates. On the other hand, Switzerland, Australia, Ireland, Iceland, Denmark, Canada, Germany, Singapore, and Italy have a higher HDI and a lower ASR DALY rate.

**Fig. 3:**
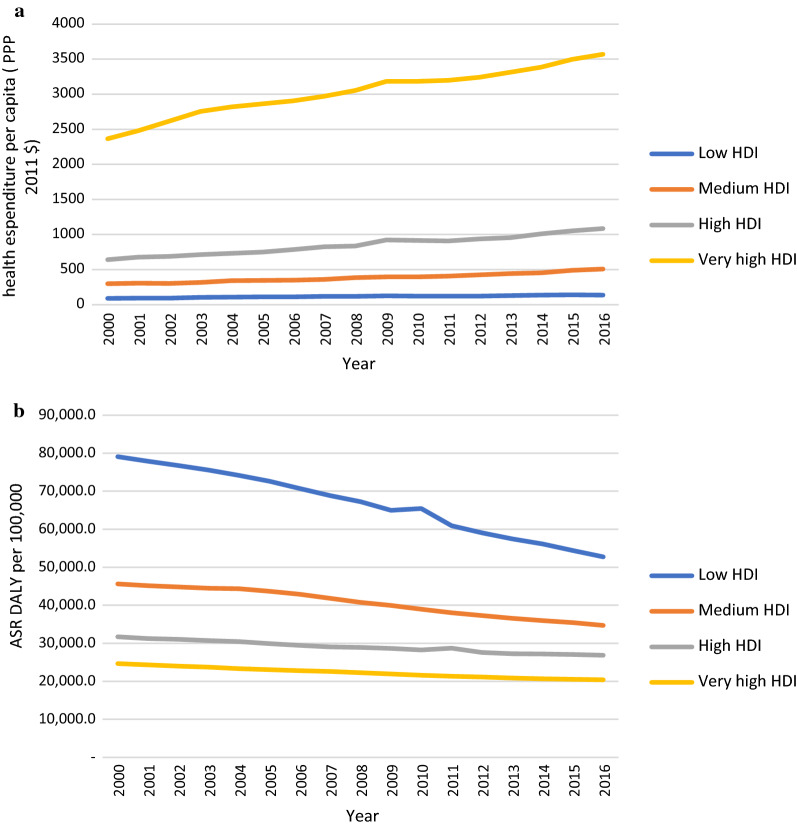
**a** Trend of health expenditure per capita (PPP $ 2011 international) by countries based on HDI. **b** ASR DALY rate per 100,000 due to all causes by countries from 2000 to 2016

Figure 3 shows HE per capita and ASR DALY trend between 2000 and 2016 in countries by HDI category. Countries with low HDI and very high HDI had the same increase on the HE per capita (about 51%) from 2000 to 2016, while the different decreases have on ASR DALY per 100,000 population (33% for low HDI and 17% for very high HDI).

The regression model results are presented in Table 1. There was a significant negative association between the logarithm of HE per capita and the logarithm of the ASR DALY rate. One percent increase in HE per capita was associated with an average of 0.28, 0.24, 0.18, and 0.27 percent reduction in ASR DALY (per 100,000 population) in low HDI, medium HDI, high HDI, and very high HDI countries, respectively (Models 1 to 4). Furthermore, an overall 1% increase in HE per capita was associated with a 0.24% reduction in ASR DALY (Model 5).

**Table 1:** Results from fixed-effect regression models

Models	Dependent variable: Log ASR DALY per 100,000				
	Model 1: low HDI countries	Model 2: Medium HDI countries	Model 3: High HDI countries	Model 4: Very high HDI countries	Model 5: All countries
Constant	12.35* (0.082)	11.96* (0.077)	11.47* (0.058)	12.13* (0.082)	11.97* (0.043)
Log HE per capita	− 0.28* (0.017)	− 0.24* (0.013)	− 0.18* (0.008)	− 0.27* (0.01)	− 0.24* (0.006)
n	49	37	49	41	176

() standard error, * significance level at 0.05

### Cost per DALY averted

**Fig. 4:**
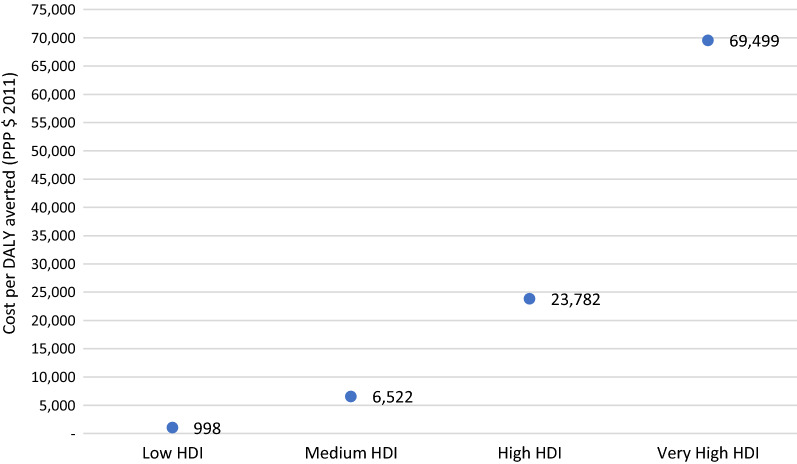
The average cost per DALY averted (PPP $) in countries according to the HDI category

The estimated cost per DALY averted was $998, $6522, $23,782, and $69,499 in low HDI, medium HDI, high HDI, and very high HDI countries, respectively (Fig. 4).

**Fig. 5:**
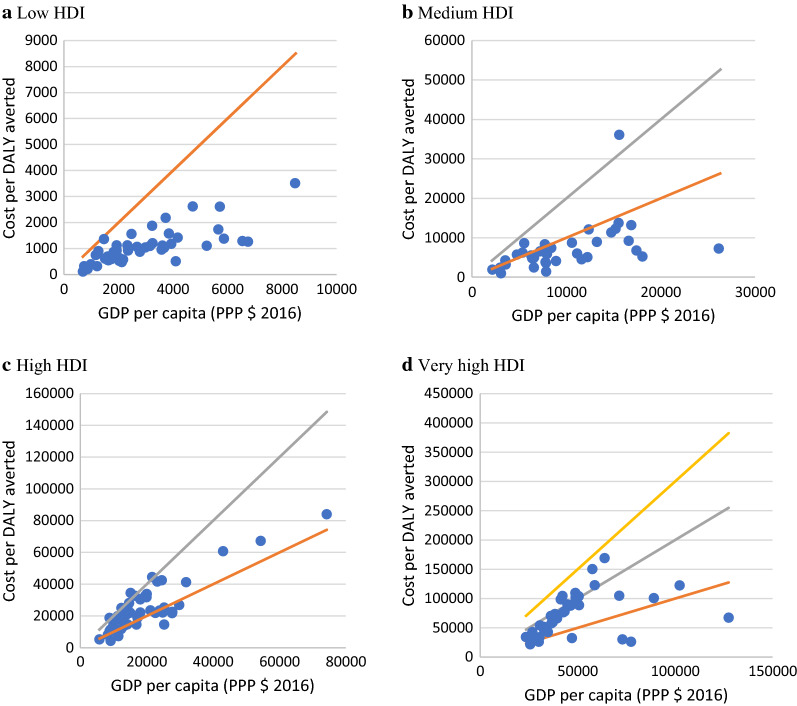
Correlation between cost per DALY averted and GDP per capita (PPP $ 2016).  1× GDP per capita,  2× GDP per capita,  3× GDP per capita

The estimated cost per DALY averted was between $109 and $3507 in low HDI countries, between $997 and $36,091 in medium HDI, between $4245 and $83,997 in high HDI, and between $21,509 and $168,720 in very high HDI countries. On average, the cost per DALY averted was 0.34 times the GDP per capita (PPP $ 2016) in low HDI countries. In contrast, it was 0.67, 1.22, and 1.46 times the GDP per capita in medium HDI, high HDI, and very high HDI countries, respectively (Fig. 5).

## Discussion

In this study, we assessed the trend of health expenditure and DALY in 176 countries over 17 years (2000–2016) and estimated the average cost per DALY averted for different countries. There was a nonlinear negative relationship between per capita health expenditure (PPP) and ASR DALY. Globally on average, a percent increase in per capita HE (PPP) was associated with a 0.24% decrease in the ASR DALY (per 100,000).

According to our findings, the per capita HE increased almost in all countries, and DALY rates decreased from 2000 to 2016. However, the increase in per capita HE in countries with a higher HDI level has been more than those with a lower HDI level, while the decrease in DALY rates in countries with a higher HDI level has been less than those with lower HDI level. This result shows that the cost per DALY averted in high HDI countries has been higher than in countries with low HDI. The average cost per DALY averted in countries with very high HDI was about 70 times higher than low HDI countries ($69,499 vs $998). In other words, on average, it costs about $70,000 to avoid one DALY in very high HDI countries, while in low HDI countries, about 70 DALYs can be avoided with this cost. This might be mainly because of the diminishing marginal return law, which means that, by the increased level of health, the marginal product of health expenditure is normally reduced [14]. The level of health in high-income countries with high HDI is significantly higher than in low HDI countries. In low HDI countries, the ASR DALY (per 100,000 population) was about 52,709 in 2016, while it was about 20,379 in very high HDI countries. For more comparability of the results across countries, it is straightforward to use a weighted DALY based on inequality. That could adjust the number of DALYs in all countries. This could be a subject for upcoming research.

Although our estimated cost per DALY averted might not be exactly used as the cost-effectiveness thresholds, these estimates could be used as a guide for determining the thresholds. For example, according to our findings in low HDI countries, it costs about $1000 to avoid one DALY and this cost was about 0.34 of the GDP per capita of these countries, so if the ICER of a new intervention was above $1000 or was more than 0.34 of the GDP per capita, that intervention is less likely to be cost-effective. Furthermore, we found that with increasing the HDI and GDP per capita, the ratio of cost per DALY averted to GDP per capita increased. For example, in low HDI countries with an average of $2955 GDP per capita (PPP) in 2016, the average cost per DALY averted was 0.34 of the GDP per capita while it was 1.46 of the GDP per capita in very high HDI countries with an average of $47,590 GDP per capita.

Different cost-effectiveness thresholds have been suggested and used in healthcare. In most studies conducted in LMICs, the “WHO-CHOICE threshold” of 1–3× GDP per capita has been widely cited as criteria for cost-effectiveness [15, 16]. According to the WHO-CHOICE threshold, interventions are considered highly cost-effective, cost-effective, and not cost-effective if their ICER is less than 1× GDP per capita, less than 3× GDP per capita, and 3× GDP per capita or higher, respectively [6, 17].

Although decision-making agencies and international organizations cited the thresholds proposed by WHO in LMICs [16], some concerns and criticisms were raised about it in recent years [2, 5, 15, 18, 19]. The previous studies, such as our study, have shown that the estimated cost-effectiveness thresholds based on opportunity cost are much lower than the 1 to 3 times GDP per capita [2, 15]. Ochalek et al. estimated the cost-effectiveness thresholds for LMICs. The results indicated that cost-effectiveness thresholds based on health opportunity cost tend to be below the lower bound suggested by WHO of 1× GDP per capita [15]. In a similar study, Woods et al. estimated the cost-effectiveness threshold based on opportunity cost for some countries with different income levels. They estimated the cost-effectiveness threshold of less than 1× GDP per capita for all studied countries [2]. These findings indicated that the thresholds suggested by the WHO might be relatively high, especially for low-income countries. Thus, in line with some previous studies, our findings suggest that the cost-effectiveness thresholds should be set at a lower level than thresholds suggested by the WHO, especially in LMICs. It should be noted that our estimate of the cost per DALY averted is according to the mean of numbers DALY. Therefore, it is not easy to compare our results with the thresholds proposed by WHO. Overall, the cost-effectiveness threshold is for new interventions. Usually, a cost per DALY averted for new interventions would be much higher than the average.

According to our results, the efficiency of the health system in some countries is low compared to other countries. Some countries were almost similar in terms of the ASR DALY but were relatively different in terms of HE per capita or vice versa. Similar results were obtained in other studies [20–23].

This study is the first research to estimate the cost per DALY averted for all countries based on GBD study results. The results can be used as a guide to determine the cost-effectiveness thresholds, especially in LMICs. However, this study has some limitations. First, for examining the effects of variables on the DALY number, we only applied the health expenditure as an independent variable, while studies have shown that different factors might affect the health outcomes [24, 25]. Adding other variables to our model can reduce the coefficient of health expenditure; subsequently, the cost per DALY averted increases. To minimize the effect of this limitation, we divided countries according to HDI and then conducted the analysis. HDI is a statistic composite index of life expectancy, education, and per capita income indicators [26]. Second, to estimate the cost per DALY averted, we used a panel regression coefficient and 2016 HE per capita data. Some countries have an inefficient healthcare system, so they incur higher expenditures for each DALY averted. These countries would achieve more remarkable results with increased efficiency in the health system. This can make up an underestimation of cost per DALY averted. Because the effect of this restriction is contrary to the last limitation, so our estimates of cost per DALY averted are constant.

## Conclusion

This study estimated for the first time the cost that is currently spent for a DALY averted based on the data from the global burden of diseases study for all countries. Overall, this study suggests that in low and medium HDI countries, the cost-effectiveness thresholds might be lower than the GDP per capita. In high and very high HDI countries, the threshold could be between one and two times the GDP per capita.

## Data Availability

The preliminary data for the analysis in this study are available in the IHME site.

## Abbreviations

DALY: Disability-adjusted life-year
HDI: Human Development Index
GDP: Gross Domestic Product
HE: Health expenditure
ASR: Age standardized rate
CEA: Cost-effectiveness analysis
ICER: Incremental cost-effectiveness ratio
QALYs: Quality-adjusted life years
GBD: Global Burden of Disease
IHME: Institute for Health Metrics and Evaluation
WHO: World Health Organization
